# Effect of trimethoprim-sulphamethoxazole on the risk of malaria in HIV-infected Ugandan children living in an area of widespread antifolate resistance

**DOI:** 10.1186/1475-2875-9-177

**Published:** 2010-06-23

**Authors:** Anne F Gasasira, Moses R Kamya, Edwin O Ochong, Neil Vora, Jane Achan, Edwin Charlebois, Theodore Ruel, Fredrick Kateera, Denise N Meya, Diane Havlir, Philip J Rosenthal, Grant Dorsey

**Affiliations:** 1 School of Medicine, Makerere University Kampala, Uganda; 2 Department of Medicine, San Francisco General Hospital, University of California, San Francisco, USA; 3 Department of Epidemiology, University of California, Berkeley, USA

## Abstract

**Background:**

Daily trimethoprim-sulfamethoxazole (TS) protects against malaria, but efficacy may be diminished as anti-folate resistance increases. This study assessed the incidence of falciparum malaria and the prevalence of resistance-conferring *Plasmodium falciparum *mutations in HIV-infected children receiving daily TS and HIV-uninfected children not taking TS.

**Materials and methods:**

Subjects were 292 HIV-infected and 517 uninfected children from two cohort studies in Kampala, Uganda observed from August 2006 to December 2008. Daily TS was given to HIV-infected, but not HIV-uninfected children and all participants were provided an insecticide-treated bed net. Standardized protocols were used to measure the incidence of malaria and identify markers of antifolate resistance.

**Results:**

Sixty-five episodes of falciparum malaria occurred in HIV-infected and 491 episodes in uninfected children during the observation period. TS was associated with a protective efficacy of 80% (0.10 vs. 0.45 episodes per person year, p < 0.001), and efficacy did not vary over three consecutive 9.5 month periods (81%, 74%, 80% respectively, p = 0.506). The prevalences of *dhfr *51I, 108N, and 59R and *dhps *437G and 540E mutations were each over 90% among parasites infecting both HIV-infected and uninfected children. Prevalence of the *dhfr *164L mutation, which is associated with high-level resistance, was significantly higher in parasites from HIV-infected compared to uninfected children (8% vs. 1%, p = 0.001). Sequencing of the *dhfr *and *dhps *genes identified only one additional polymorphism, *dhps *581G, in 2 of 30 samples from HIV-infected and 0 of 54 samples from uninfected children.

**Conclusion:**

Despite high prevalence of known anti-folate resistance-mediating mutations, TS prophylaxis was highly effective against malaria, but was associated with presence of *dhfr *164L mutation.

## Background

Daily prophylaxis with the anti-folate combination trimethoprim-sulphamethoxazole (TS) has been shown to reduce morbidity and improve survival in HIV-infected populations [1,2]. In addition to the prevention of opportunistic and other common bacterial infections associated with HIV infection, TS reduces the risk of malaria in HIV-infected adults and children living in sub-Saharan Africa [1,3-5]. The World Health Organization, UNAIDS and UNICEF recommend TS prophylaxis for all HIV-infected patients with symptomatic disease (WHO Stage 2, 3, and 4), Stage 1 HIV-infected adults with CD4 lymphocyte counts less than 350 cells/μl, Stage 1 HIV-infected children with CD4 lymphocyte percentage less than 25%, and all infants born to HIV-infected mothers (HIV-exposed) [6].

Despite the evidence for significant benefits associated with TS use in HIV-infected patients, implementation of TS prophylaxis in sub-Saharan Africa remains low. A recent UNICEF report estimated that only 4% of the 4 million children in need of TS prophylaxis in sub-Saharan Africa are receiving this intervention [7]. There are concerns that in areas where anti-folate resistance is extensive, TS may not be effective in preventing malaria and bacterial infections, while in areas where resistance is not yet widespread, expanded TS use may lead to the rapid selection and spread of *Plasmodium falciparum *parasites resistant to anti-folates. This in turn may diminish the preventive efficacy of TS and sulphadoxine-pyrimethamine (SP), which acts against the same two enzyme targets as TS. Cross-resistance between TS and SP is of particular concern as, although SP is being replaced by more effective agents for the treatment of malaria, it remains the only recommended drug for intermittent preventive therapy (IPT), a validated measure to prevent malaria in pregnant women [8].

To characterize the risks and benefits of daily TS, this study compared the incidence of malaria and the prevalence of anti-folate resistance-conferring mutations in *P. falciparum *infections in HIV-uninfected children not taking TS and HIV-infected children taking daily TS in an area of moderate malaria transmission. Further, the effect of time on the protective efficacy of TS and the prevalence of anti-folate resistance-conferring mutations was evaluated.

## Methods

### Study site

The study was carried out in Kampala, Uganda. Kampala is an urban setting in south-central Uganda where malaria is mesoendemic, occurring perennially with peaks following two rainy seasons, and an entomological inoculation rate (EIR) estimated to be less than 10 infective bites per person year in 2005 (unpublished data).

### Study subjects

Subjects were children participating in two parallel cohort studies, which have been described previously [4,9] and are summarized here briefly. The first study enrolled 599 healthy children presumed to be HIV-uninfected (432 tested HIV negative, 167 HIV test results unavailable; all subjects were healthy) into a community-based cohort from a geographically defined census population using probability sampling from November 2004 through April 2005. Eligibility criteria included: 1) age 1-10 years, 2) agreement to come to the study clinic for any febrile episode or other illness, 3) agreement to remain in Kampala for the duration of the study, 4) agreement to avoid medications administered outside the study protocol, 5) lack of history of any known serious chronic disease requiring frequent medical attention, including HIV/AIDS, 6) weight >10 kg, and 7) provision of informed consent from parent or guardian. None of the HIV-uninfected cohort study participants were prescribed TS prophylaxis. All study participants were given an insecticide treated bed net (ITN) between May-June 2006. The second study enrolled 300 HIV-infected children from a pediatric HIV clinic in Kampala from October 2005 through August 2006 using convenience sampling. Eligibility criteria were the same as for the HIV-uninfected cohort, except for: 1) living within a 20 km radius of the study clinic, 2) no restriction based on a history of serious chronic disease, and 3) weight >5 kg. All HIV-infected cohort participants were prescribed daily TS and provided with an ITN at enrollment.

### Study participant follow-up

Similar protocols were used for follow-up of both cohorts. Subjects were followed for all of their medical problems in dedicated study clinics open seven days a week, with after hours care available at Mulago Hospital. Children who presented with new medical problems underwent standardized medical evaluation. A set of algorithms was developed to guide therapy for common illnesses. Medications with anti-malarial activity were avoided for the treatment of non-malarial illnesses when possible. HIV-infected subjects meeting standard WHO eligibility criteria were provided antiretroviral therapy according to local guidelines[10]. The preferred first line therapy for those initiated to ART was Efavirenz (EFV), lamivudine (3TC) and zidovudine (AZT). Subjects who presented to the clinics with a documented fever (tympanic temperature >38.0°C) or history of fever in the previous 24 hours had blood obtained by fingerprick for a thick blood smear. If the thick blood smear was positive, the patient was diagnosed with malaria regardless of the parasite density. HIV-uninfected participants diagnosed with uncomplicated malaria were assigned randomly to anti-malarial treatment with sulphadoxine-pyrimethamine plus amodiaquine (SP + AQ), amodiaquine plus artesunate (AQ + AS), or artemether-lumefantrine (AL). HIV-infected children were treated with AQ + AS between August 2006 and June 2007. Use of AQ + AS in this study was stopped after a high risk of neutropaenia secondary to malaria treatment was observed [11]; it was replaced with AL from July 2007 to the end of the study. All episodes of complicated malaria and treatment failures occurring within 14 days of initial therapy were treated with quinine.

TS adherence in HIV-infected participants was measured during routine monthly visits using caregivers' self-report of administering drugs during the previous 3 days.

Study participants were withdrawn from follow-up if any of the following occurred: 1) movement out of study area for >60 consecutive days, 2) inability to be located for >60 consecutive days, 3) withdrawal of informed consent, or 4) inability to adhere to study schedule and procedures.

### Laboratory methods

Thick smears were stained with 2% Giemsa for 30 minutes. Parasite density was estimated by counting the number of asexual parasites per 200 white blood cells and calculating parasites per μL, assuming a white blood cell count of 8,000 cells per μL. A smear was judged to be negative if no parasites were seen after review of 100 high-powered fields. Final microscopy results were based on a rigorous quality control system which included re-reading all blood smears by a second microscopist and resolution of any discrepancies between the first and second readings by a third microscopist. Parasite species on the day malaria was diagnosed was determined using nested PCR of 18 S small sub-unit ribosomal DNA as previously described [12].

For repeat episodes of malaria in the same study participant, molecular genotyping was used to distinguish new from recrudescent infections. DNA was isolated from blood samples collected on filter paper on the day of malaria diagnosis, and samples were genotyped in a stepwise fashion by assessing polymorphisms in *msp-2*, *msp-1*, and four microsatellites as previously described [13]. If, for any of the six loci, an allele was not shared between consecutive episodes of malaria, the episode was classified as a new infection. If at least one allele was shared between consecutive episodes of malaria at all six loci, the episode was classified as a recrudescence.

Samples were tested on the day malaria was diagnosed for the following polymorphisms associated with anti-folate resistance: dihydrofolate reductase (*dhfr*) N51I, C59R, S108N, and I164L; and dihydropteroate synthetase (*dhps*) A437G and K540E. Polymorphisms were identified using a nested polymerase chain reaction followed by sequence-specific restriction enzyme digestion. Primers, amplification conditions, and restriction endonucleases for these assays were as previously described [14,15]. Digestion products were visualized by agarose gel electrophoresis and results were classified according to the migration patterns of the fragments. Laboratory investigators were blinded to clinical data at the time of molecular analysis.

For a subset of samples, the complete *dhfr *and *dhps *genes were amplified by nested PCR and sequenced. For *dhfr*, the gene was amplified with primers 3A (5'-CCCAAATAGCTAGTTCAGGGGAAC and 4B (5'-TCATCTTCTTCTTCATCATCATCATC) followed by the nested primers 3'A (5'-CTCCTTTTTATGATGGAACAAGTCTGCG) and 3B (5'-TGACATGTATCTTTGTCATCATTC) to yield a 785 bp fragment.

For *dhps*, primers were 6A (5'-GGATCAGAAGATGAATAATCATGT) and 3B (5'-CACTAAACCATTAGAGTACTTGAC), followed by 7A (5'-CCATTCCTCATGTGTATACAACAC) and 5B (5'-GTTTAATCACATGTTTGCACTTTC-3'), yielding a 1326 bp fragment.

Amplicons were purified using Exosap (USB) and sequenced bi-directionally using nested primers at the Genomics Core Facility, UCSF. Sequence data were aligned and manually curated using Lasergene (DNASTAR).

### Statistical analysis

The period of observation began on August 1, 2006 (when enrollment for both cohorts was complete and all study participants had been given an ITN) and continued through December 15, 2008 (when follow-up for the HIV-uninfected cohort ended) or the date of premature study withdrawal.

Data were entered and verified using Access (Microsoft Corporation, Redmond, WA). Analyses were performed using STATA version 10.0 (Stata Corp., College Station, TX). Log-transformed parasite densities were compared using the two-sample t-test and categorical variables (comparisons of mutation prevalence and parasite species) were analysed using Fisher exact or chi-square tests. Malaria incidence was defined as the number of new episodes of malaria (recrudescences were excluded) per period of observation. Negative binomial regression models were used to measure the association between use of TS prophylaxis and malaria incidence (expressed as an incidence rate ratio (IRR)), controlling for age. The protective efficacy of TS was defined as 1 - IRR. Tests for trend were used to assess for changes in the protective efficacy of TS and the prevalence of anti-folate resistance-conferring mutations over the following time periods: August 1 2006 to May 15 2007; May 16 2007 to February 29 2008 and March 1 2008 to December 15 2008. A p-value < 0.05 was considered statistically significant.

### Ethical approval

Written informed consent was obtained from the parents/guardians of children for their participation in the study. Ethical approval for the two cohort studies was obtained from the Uganda National Council of Science and Technology, Makerere University Research and Ethics Committee, and the University of California, San Francisco Committee on Human Research.

## Results

### Study participants

Characteristics of study participants are shown in Table 1. Of the 599 participants enrolled into the HIV-uninfected cohort, 517 were still in active follow-up by August 2006 and included in the study. Likewise, 292 of the 300 HIV-infected children initially enrolled were active and included in the present study. The median level of TS adherence in the HIV-infected population over the study period was 100%, with only 216 of 8257 (3%) assessments associated with less than 100% adherence. The mean age at the beginning of the observation period was significantly higher for HIV-uninfected compared to HIV-infected participants (7.4 vs. 5.7 years, p < 0.0001), primarily because the HIV-infected cohort was recruited about one year later than the uninfected cohort. A total of 452 (87%) HIV-uninfected children and 277 (94%) HIV-infected children were followed for the full observation period. The mean CD4 cell percentage in the HIV-infected cohort was 23% at baseline, 76 (26%) participants received anti-retroviral therapy (ART) throughout the study period, and 65 (22%) were initiated on ART during follow-up.

**Table 1:** Patient characteristics

Characteristics	HIV-uninfected*	HIV-infected*
Number of subjects	517	292
Total person years of observation	1095	665
Median duration of follow up in yrs (range)	2.1(0.2-2.4)	2.4 (0-2.4)
Mean age in yrs at the start of follow-up (SD)	7.4 (2.7)	6.0(2.6)
Mean CD4 percent at start of follow-up (SD)	N/A	23%(8.6)
Antiretroviral (ART) use during follow-up		
Total person years on ART	N/A	275
Number of participants on ART at start of follow-up	N/A	76 (26%)
Number of participants initiated on ART during follow-up	N/A	65 (22%)

*All HIV-uninfected children were not receiving TS while all HIV-infected children were receiving daily TS

### Characteristics of malaria episodes

The large majority of treatments for malaria were for new, rather than recrudescent infections, as expected for cohorts in which malaria episodes were treated with highly efficacious combination regimens (Table 2). Ninety-two percent of new malaria episodes were due to *P. falciparum *in both HIV-uninfected and HIV-infected children. The geometric mean parasite density of malaria infections was lower for HIV-infected (6,462 parasites/μl) compared to HIV-uninfected (11,270 parasites/μl) children, however this difference was not statistically significant (p = 0.397).

**Table 2:** Characteristics of malaria episodes

Characteristics	HIV-uninfected* (n = 517)	HIV-infected* (n = 292)
Number of treatments for malaria	511	65
New episodes of malaria^†^	491	65
Species for new episodes		
*P. falciparum*^‡^	451 (92%)	60 (92%)
*P. malariae*	23 (4%)	2 (3%)
*P. ovale*	14 (3%)	3 (5%)
*P. vivax*	3 (1%)	0
Geometric means parasite density per μL^§^	11230	6462

* All HIV-uninfected participants were not receiving TS and all HIV-infected participants were receiving daily TS

^† ^After exclusion of episodes due to recrudescences identified by genotyping

^‡ ^Includes *P. falciparum *mono and mixed infections

^§ ^Only include new episodes of falciparum malaria

### Malaria incidence

The protective efficacy of TS for the entire observation period was 80% (95% CI 72-85%) adjusting for age at the start of the study, and efficacy did not decline over the 3 consecutive 9.5-months periods that constituted the observation period [IRRs: 0.19 (0.12-0.28), 0.26 (0.15-0.43) and 0.20 (0.10-0.38), respectively (Table 3)]. The protective efficacy of TS was similar for HIV-infected participants receiving [76% (95% C.I. 63-84%)] and not receiving ART [83% (95% C.I 74-89%)].

**Table 3:** Comparison of malaria incidence

Time Period	HIV-uninfected			HIV-infected			IRR (95% C.I)
	Episodes	Person time (yrs)	Incidence	Episodes	Person time	Incidence	
Aug 1^st ^06 - Dec 15^th ^08	491	1095	0.45/psnyr	65	665	0.10/psnyr	0.20 (0.15-0.28)
Aug 1^st ^06 - May 15^th ^07	267	397	0.67/psnyr	28	224	0.13/psnyr	0.19 (0.12-0.28)
May 16^th ^07 - Feb 29^th ^08	132	364	0.36/psnyr	23	222	0.10/psnyr	0.26 (0.15-0.43)
Mar 1^st ^08 - Dec 15^th ^08	92	334	0.28/psnyr	14	220	0.06/psnyr	0.20 (0.10-0.38)

*Incident Rate Ratio controlling for age at beginning each observation period

### Prevalence of resistance-mediating polymorphisms

In new *P. falciparum *infections, the prevalences of five *dhfr *and *dhps *point mutations known to mediate diminished response to therapy with SP and to be common in East Africa [16] (*dhfr *51I, 108N, and C59R and *dhps *437G and 540E) were all over 90%, independent of TS use (Table 4) and time. The simultaneous presence of all 5 of these mutations was also common for HIV-uninfected and infected participants (86% vs. 92%, p = 0.222) and prevalence did not increase significantly over the 3 consecutive 9.5-month periods in HIV-infected (96%, 86%, 93% respectively, p = 0.446) or uninfected (82%, 89%, 88% respectively, p = 0.467) participants.

**Table 4:** Prevalence of molecular markers of anti-folate resistance among new episodes of *P. falciparum *malaria

Characteristics	HIV-uninfected* (n = 352)	HIV-infected* (n = 60)	P-value
Prevalence of *dhfr *mutations			
51I	179(99%)^†^	60(100%)	0.563
59R	163 (91%)^†^	56(93%)	0.510
108N	180(100%)^†^	60 (100%)	
164L	1 (1%)^†^	5 (8%)	0.001
			
Prevalence of *dhps *mutations			
437G	172 (96%)^†^	58 (97%)	0.709
540E	174(97%)^†^	59 (99%)	0.506
Quintuple mutation^‡^	154 (86%)^†^	55 (92%)	0.222

*All HIV-uninfected participants were not receiving TS and all HIV-infected participants were receiving daily TS

^† ^Assessed in a random sample of 180 episodes matched by malaria treatment and calendar time

^‡ ^*dhfr *51I/59R/108N + *dhps *437G/540E mutations

In contrast to the mutations noted above, *dhfr *164L, which has been associated with high-level SP resistance, was uncommon. Of note, this mutation was significantly more common in HIV-infected compared to HIV-uninfected participants (8% vs. 1%, p = 0.001). As with the other mutations, prevalence of the *dhfr *164L mutation did not change with time in parasites from HIV-infected (12%, 10%, 0% p = 0.417) or uninfected participants (0%, 2%, 0%, p = 0.375).

To assess for novel mutations in the *dhfr *or *dhps *genes as a result of daily TS use, the complete genes were sequenced in a subset of samples, including *dhfr *from 40 and *dhps *from 30 HIV-infected children receiving TS and *dhfr *from 73 and *dhps *from 54 uninfected children not receiving TS. Sequencing confirmed the sequences at known polymorphic alleles in all samples but one, in which *dhfr *108N/51I/164L mutations were seen in the initial analysis and 108N/51I/59R seen with sequencing; presumably the discrepancy was due to characterization of different strains in a mixed infection. Sequencing identified only one additional polymorphism, the previously reported *dhps *581G mutation, which was seen in samples from 2 of 30 children receiving daily TS and none of the 54 HIV-uninfected children for whom sequence results were available (p = 0.125).

## Discussion

The presence of simultaneous cohorts including HIV-infected children receiving daily TS and HIV-uninfected children not receiving TS allowed for the assessment of the anti-malarial preventive efficacy of TS. Results from this study show that daily TS was highly efficacious in conferring protection against malaria in HIV-infected children despite a high prevalence of resistance-mediating *P. falciparum *polymorphisms. This study adds to a previous one by this group which reported a 97% reduction in malaria risk in HIV-infected children associated with the combined use of TS and ITNs [4]. In the present study, without the confounder of ITN use in only the HIV-infected children, the protective efficacy of daily TS was more clearly defined, and it remained high.

TS use has recently been shown to protect against malaria in adults [3] and children [4] in Uganda, but it has not been clear if continued protection will be seen with increasing prevalence of mutations in the target enzymes *dhfr *and *dhps *that limit anti-malarial treatment efficacy of the related anti-folate SP [17-20]. However, in the present study, despite some of the highest prevalences of 5 key *dhfr *and *dhps *mutations reported in Africa, TS offered strong protective efficacy against malaria, and protective efficacy was maintained throughout the 29-month observation period.

Drugs that inhibit the folate pathway enzymes *dhfr *and *dhps *have important roles in the treatment of many infections. TS is active against many bacteria and opportunistic pathogens, leading to recommendations for its widespread use in HIV-infected and exposed individuals in Africa. SP, which inhibits the same two enzymes as TS, has been a standard anti-malarial therapy in Africa. Use of SP to treat malaria is now discouraged due to increasing resistance, especially in Southern and East Africa, with resistance mediated by step-wise selection and also selective sweeps of 5 key mutations [21]. Additional mutations in *dhfr *and *dhps*, notably *dhfr *164L, mediate higher levels of resistance, but these mutations are uncommon in Africa [22]. Despite decreasing malaria treatment efficacy, SP retains a key role in malaria control, as it is the only drug well-established to protect against malaria when used as IPT in pregnant women [23] or infants [24]. In summary, the anti-folates TS and SP remain critical elements of control efforts for HIV infection and malaria, but resistance-mediating mutations are common in *P. falciparum *in many areas, and key questions remain inadequately answered. This study considered the following three major concerns regarding daily use of TS in HIV-infected individuals.

First, did daily TS offer protection against malaria despite the presence of anti-folate resistance-mediating mutations? Five key resistance-mediating mutations, which were already known to be common in much of East Africa [25], were remarkably common in parasites isolated from infected patients in this study. In this setting, daily TS nonetheless offered strong preventive efficacy against malaria. Thus, although the resistance-mediating mutations may have mitigated the protective efficacy of TS to some extent, the intervention nonetheless had a pronounced effect. This result bodes well for Africa, where the prevalence of these 5 key mutations is increasing, but other mutations that mediate higher-level resistance remain uncommon.

Second, did TS use select for parasites with additional anti-folate resistance mediating mutations that might ablate any protective efficacy of anti-folates? Blood samples were screened for the *dhfr *164L mutation, which is common in parts of South America and Asia [21], but has not shown high prevalence in Africa. Recently, however, the mutation was seen in 4% of parasites isolated in Western Kenya [26], in 4.7% of parasites in Malawi from subjects receiving SP [27], and 4-14% of symptomatic subjects not receiving anti-folates in two low transmission highland regions of Western Uganda [28]. In this study *dhfr *164L was uncommon in HIV-uninfected children who did not receive TS. However, compared to those who did not receive the intervention, prevalence of the *dhfr *164L mutation was significantly greater in HIV-infected children who received TS. These results indicate that, in certain settings, daily TS may select for *dhfr *164L. The clinical significance of selection for this additional mutation is unknown, but biochemical studies have shown profound resistance in parasites harboring *dhfr *164L [29]. However, it remains unclear whether the *dhfr *164L mutation will spread in Africa. Indeed, as is the case with some other resistance-mediating polymorphisms that may engender a fitness cost (e.g. increased copy number of the putative drug transporter gene *pfmdr1*), the high level of anti-malarial immunity of populations in areas of high malaria transmission in Africa may limit selection for *dhfr *164L [18]. The complete *dhfr *and *dhps *genes were sequenced in a subset of samples and only one additional polymorphism, the *dhps *581G mutation, was identified in 2 samples from children receiving daily TS. This mutation has been associated with high-level in vitro sulphadoxine resistance in *P. falciparum *[30], has been seen in Southeast Asia and South America [30,31], and had a prevalence of 55% in a recent study in northern Tanzania [32]. In Kampala, the prevalence of the 581G mutation was low, and it is unclear if it was selected by daily TS. Importantly, no novel *dhfr *or *dhps *mutations were identified.

Third, did the protective efficacy of TS decline over time? In this study, TS efficacy was maintained over the 29-month observation period. This is not surprising given the stability of the anti-folate-mediating mutations assessed. Five of the 6 mutations evaluated were close to saturated throughout the study period, while the prevalence of the 164L mutation, which is not yet widespread, did not increase in those exposed or unexposed to TS. The evolution of anti-folate resistance likely occurred over several years, limiting the ability of the study to detect changes over the relatively short observation period. Therefore, while the present study gives an indication that TS prophylactic efficacy may be sustained in areas where background anti-folate resistance is extensive, more studies will be needed to assess its longer-term efficacy.

This study had some limitations. For ethical reasons, HIV-infection and TS use could not be separated. Therefore, comparisons of TS-unexposed and exposed participants were between HIV-uninfected children not receiving TS and HIV-infected children receiving the intervention. Sixteen percent of presumed HIV-uninfected subjects were not tested for HIV, as they had either been excluded from the cohort prior to testing or declined to be tested. However, the lack of HIV infection among the 432 children who were tested suggests that very few of the cohort, if any, had this infection, and any bias resulting from misclassification of HIV status would be expected to underestimate differences seen between the HIV-infected and HIV-uninfected cohorts. The impact of TS on 6 well characterized polymorphisms in *dhfr *and *dhps *was considered in all samples, but due to logistical constraints whole gene sequences were assessed in only a subset of samples chosen based on amplification and sequencing success; sequencing of only a portion of the total might have introduced bias, and so it is possible that other rare polymorphisms were missed. Finally, the study was done in a relatively low transmission setting, and findings may not be generalizable to other settings, particularly those with higher malaria transmission intensity. These limitations, however, do not alter the key conclusions of the study, as noted above.

## Conclusions

Considering impacts on malaria, this study strongly supports the use of TS prophylaxis in HIV-infected children in Africa. Further, given the excellent protective efficacy of TS, consideration might be given for its use in populations at highest risk of adverse consequences of malaria in addition to other transmission reduction measures such as ITNs. The study was not well-equipped to assess the impact of daily TS on the selection of common anti-folate resistance-mediating mutations, as these were already very common at our study site. The findings of this study, together with those from studies assessing SP efficacy for IPTp, suggest that the prophylactic efficacies of TS and SP remain high in even where anti-folate resistance is widespread. However, the selection of an additional mutation, *dhfr *164L, in children receiving daily TS is concerning, and suggests the need for continued surveillance to monitor the protective efficacy of TS and the prevalence of resistance-mediating polymorphisms.

## Competing interests

The authors declare that they have no competing interests.

## Authors' contributions

MRK, DH, PJR, GD, EC, and AFG contributed to the design of the study. MRK, JA, DM, FK, NV, and AFG supervised enrollment and follow-up of study participants and directed the clinical studies. NV and EO performed the molecular studies. MRK, DH, PRJ, TR, EC and GD participated in the oversight of study activities. GD and AFG verified and analysed the data. All authors assisted with interpretation of the data and preparation of the manuscript and approved the final report.

## About the Authors

PJR is a Doris Duke Charitable Foundation Distinguished Clinical Scientist.
